# *In vitro* activity of antibiotic monotherapy and combination therapy with bacteriophages against *Staphylococcus aureus* LVAD-driveline infections

**DOI:** 10.1128/jcm.00272-25

**Published:** 2025-10-09

**Authors:** Michèle M. Molendijk, Nelianne J. Verkaik, Corné P. de Vogel, Nicole Lemmens-den Toom, Gwenan M. Knight, Kadir Caliskan, Lonneke G. M. Bode, Annelies Verbon, Marion P. G. Koopmans, Miranda de Graaf, Willem J. B. van Wamel

**Affiliations:** 1 Department Medical Microbiology and Infectious Diseases, Erasmus MC, Rotterdam, the Netherlands; 2 Department of Viroscience, Erasmus MC, Rotterdam, the Netherlands; 3 Department of Infectious Disease Epidemiology, London School of Hygiene & Tropical Medicine 240325, London, United Kingdom; 4 Department of Cardiology, Erasmus MC, Rotterdam, the Netherlands; 5 Department of Internal Medicine, UMC Utrecht, Utrecht, the Netherlands; Universitat Munster, Münster, Germany

**Keywords:** LVAD driveline infection, biofilm, *Staphylococcus aureus*, bacteriophages, isothermal microcalorimetry

## Abstract

Left-ventricular assist devices (LVADs) are increasingly used as a bridge to heart transplantation and destination therapy. These devices, especially the driveline, are susceptible to difficult-to-treat infections, associated with high morbidity and mortality rates. *Staphylococcus aureus* (*S. aureus*) is a major causative pathogen of LVAD infections. Antibiotic resistance and biofilm formation can complicate the treatment of these infections. A novel *in vitro* assay was developed to study the antibiotic susceptibility of *S. aureus* biofilm grown on LVAD drivelines. Besides antibiotic monotherapy, the effect of various antibiotics combined with rifampicin was studied. Additionally, we explored the efficacy of four individual phages and phage-antibiotic combinations as potential treatment strategies. Our data showed a decrease of susceptibility of the *S. aureus* biofilms to antibiotic monotherapy compared to planktonic *S. aureus*. With only rifampicin and erythromycin monotherapy resulting in full bacterial clearance. Combining antibiotics with rifampicin showed similar antimicrobial efficacy against *S. aureus* biofilms as rifampicin monotherapy. While both individual phages and a phage cocktail were effective against planktonic bacteria, phage efficacy was limited against *S. aureus* in biofilm. Combining phages with antibiotics did not clearly improve treatment efficacy, compared to antibiotic monotherapy. Contrarily, it even increased bacterial growth when phage administration preceded antibiotic treatment. Here, both antibiotic- and phage monotherapy showed reduced efficacy on LVAD-driveline biofilms. Additionally, phages did not show an additive value to antibiotic treatment of LVAD driveline infections. Further studies are needed to elucidate optimal treatment strategies for LVAD-driveline infections.

**IMPORTANCE:**

Current treatment strategies for *S. aureus* LVAD-driveline infections are based on *in vitro* antibiotic susceptibility of planktonic bacteria. However, LVAD infections are most often biofilm-related, which decreases antibiotic susceptibility significantly, resulting in discrepancies between *in vitro* antibiotic susceptibility and *in vivo* treatment success. Here, we have developed a novel *in vitro* assay to determine antibiotic susceptibility of *S. aureus* biofilm, grown in conditions relevant to LVAD-driveline infections. Next to antibiotic susceptibility, the susceptibility of this biofilm to bacteriophage mono- and combination treatment with antibiotics was evaluated as an alternative treatment strategy. In the future, this assay can be used to provide a better insight in *in vivo* antibiotic- and bacteriophage susceptibility of LVAD-driveline biofilms. Thereby improving *in vivo* treatment strategies for LVAD-driveline infections.

## INTRODUCTION

Left ventricular assist devices (LVAD) serve as a bridge to heart transplantation and are increasingly used as destination therapy (1). A LVAD is a cardiac pump that is connected to an external power source via a driveline protruding from the skin (2). There is a risk of LVAD-driveline infections through translocation of microbes at the driveline exit, which can result in severe infections, such as bacteremia, pump, or cannula infections (3). LVAD infections are responsible for 15% of LVAD-related deaths 1 year after placement and occur in approximately 30% of patients (1, 2).

One of the major causative pathogens of LVAD infections is *Staphylococcus aureus*. The high rate of antibiotic resistance and biofilm formation by *S. aureus* complicate the treatment of LVAD infections (2–4). Currently, no specific guidance for antibiotic treatment of LVAD infections exists, and antibiotic choice is usually determined using *in vitro* susceptibility testing of planktonic bacteria. However, this approach can lead to discrepancies between *in vitro* susceptibility testing and antimicrobial efficacy *in vivo (5–7*), since significant differences in susceptibility have been observed not only between biofilms and planktonic cells but also between different biofilms (8, 9). Biofilm heterogeneity arises from phenotypic and adaptive variation and is influenced by various factors, such as nutrient availability and the material on which the biofilm is grown (10–13). This variation not only complicates the treatment of biofilms but also highlights the importance of experimental conditions during research aimed at understanding them (11, 12).

Bacteriophages (phages), viruses that can lyse bacteria, are a potential alternative treatment for bacterial infections (14–18). In addition, some combinations of phages with sub-lethal antibiotic concentrations can enhance antimicrobial efficacy, a phenomenon called phage-antibiotic synergy (PAS) (16, 19–24). Currently, there are no published clinical trials on phage therapy for LVAD infection; nonetheless, 20 cases of phage therapy (adjunctive to antibiotic treatment) for LVAD infections have been reported (25). Of these, 11 have resulted in favorable outcomes, while 9 were reported as treatment failures (4, 26–34). Of the 20 case reports, 9 reported on the treatment of *S. aureus* LVAD infections, describing varying outcomes ranging from complete bacterial eradication to treatment failure (26, 28, 30–33, 35). Since the set-up of treatment differed between these patients (e.g., route of administration, phages used, etc.), the treatment outcome could not be linked to specific factors. However, it is likely that the development of phage resistance, PAS, and biofilm formation all play an important role (4).

Here, a novel assay based on isothermal microcalorimetry (IMC) was developed to particularly mimic a *S. aureus* infection of LVAD drivelines. IMC is an analytical tool used to measure heat produced during chemical and physical cellular processes. The resulting increase in heat flow is considered a proxy for microbial metabolic activity, aggregation and growth (13, 36). IMC is especially suited to study biofilm-associated bacteria since the biofilm remains undisturbed and can be monitored in real-time and in many different growth conditions (8, 36). Here, IMC was used to monitor biofilm-associated bacteria in physiologically relevant conditions; with *S. aureus* biofilm grown on a LVAD-driveline carrier and in the presence of serum. This assay was used to evaluate the antimicrobial efficacy of various individual antibiotics, and in combination with rifampicin, against LVAD-driveline biofilms.

To increase the clinical relevance of this assay, a *S. aureus* strain (SA-Ld) isolated from a patient suffering from a LVAD-driveline infection was used. This strain belongs to Sequence Type (ST) 5, which is highly prevalent in human clinical samples worldwide, and is a major cause of hospital-associated infections; moreover, strains belonging to this ST are often methicillin-resistant. Additionally, ST5 strains are well known for their ability to form biofilms (37, 38). The formation of biofilms of clonal lineage 5, to which ST5 strains belong, is dependent on extracellular DNA production, which can influence antibiotic resistance (39, 40).

Next to antibiotic susceptibility, the susceptibility of *S. aureus* SA-Ld to four phages (phage K, Sb-1, ISP, and RPCSa2) was assessed, both individually and combined as a phage cocktail. These four phages were specifically selected for their ability to degrade biofilms (16, 17, 21, 41–46). Moreover, phage Sb-1, Phage K, and a phage cocktail from which phage RPCSa2 was originally isolated (PYO) have been successfully applied to treat LVAD infections *in vivo* (32, 33, 35, 47). In addition to monotherapy, the phage cocktail was also combined with antibiotics to evaluate both a simultaneous and sequential combination therapy strategies.

Altogether, this study provides insights in various treatment strategies for LVAD-driveline infections which can guide future therapy for *S. aureus* LVAD-related infections.

## RESULTS

### Antimicrobial efficacy of antibiotic mono- and combination therapy

**Fig 1:**
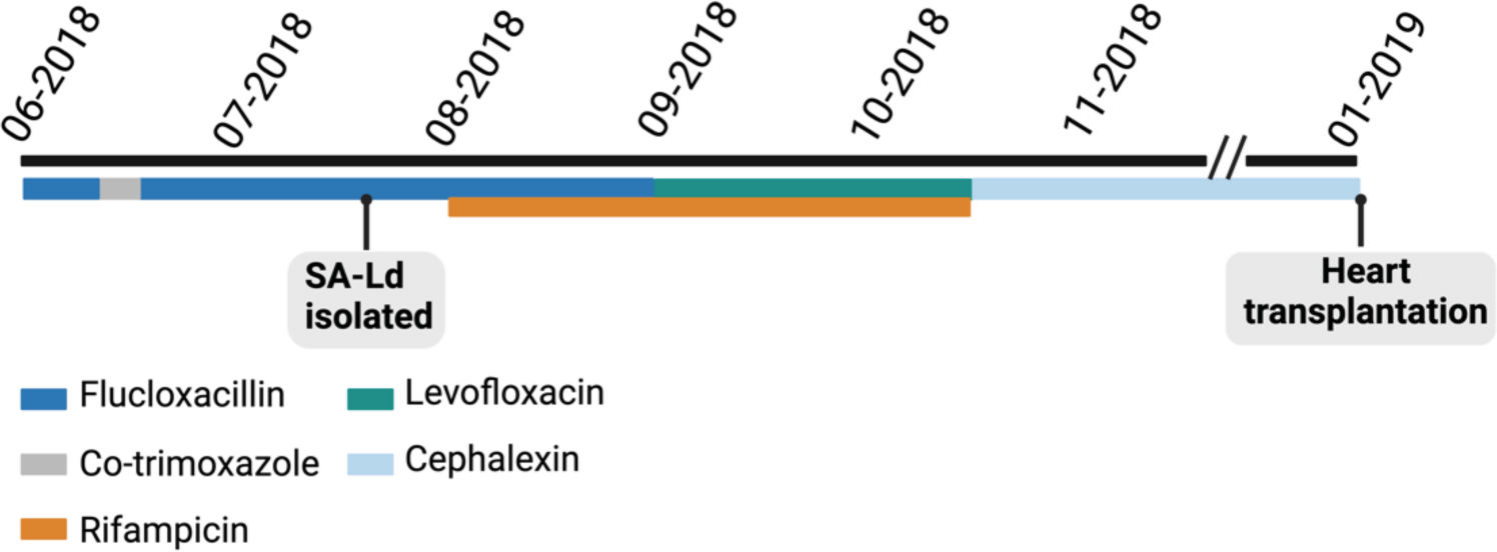
Timeline of treatments for the LVAD driveline infection. After diagnosis, treatment commenced in June 2018. The patient was initially treated with intravenous flucloxacillin (6–12 g/24 h) for 8 weeks. With a 1-week interruption during which he received co-trimoxazole orally. Subsequently, flucloxacillin was restarted intravenously and combined with rifampicin (450 mg, twice a day). Four weeks after the addition of rifampicin to the treatment regime, flucloxacillin was replaced with levofloxacin orally (500 mg, twice a day). Clinical signs and symptoms resolved and by mid-October 2018, the patient transitioned to suppressive therapy with cephalexin (1,000 mg, orally, three times a day). In January 2019, with no signs of infection, a heart transplantation was performed.

Despite belonging to ST5, which is known to include MRSA strains, SA-Ld exhibited high *in vitro* susceptibility to various antibiotics, as determined by standardized diagnostic testing (Table 1). However, despite this susceptibility, the patient required extensive treatment (Fig. 1), highlighting the challenges and complexities associated with these infections.

**TABLE 1:** Susceptibility of the *S. aureus* patient isolate SA-Ld to a range of antibiotics

Antibiotic	Interpretation according to EUCAST	VITEK MIC (µg/mL)^*a*^
Flucloxacillin^*b*^	S	–^*c*^
Cefuroxime	S	–
Gentamicin	S	≤0.5
Co-trimoxazole^*b*^	S	≤0.5
Levofloxacin^*b*^	S	≤0.25
Erythromycin	S	≤1
Clindamycin	S	≤0.25
Vancomycin	S	≤1
Rifampicin^*b*^	S	≤0.03
Fusidic acid	S	≤0.5
Linezolid	S	≤4
Mupirocin	S	≤1
Doxycycline	S	≤1

^*a*^ MIC, minimal inhibitory concentration. If no MIC is mentioned, susceptibility is determined by disk diffusion cefoxitin.

^*b*^ Antibiotic used to treat the patient.

^*c*^ –, not performed.

**Fig 2:**
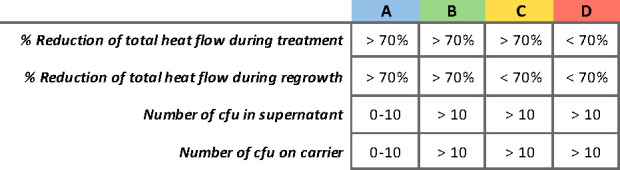
Overview of the four categories of antimicrobial efficiency. Antimicrobial efficacy categories are based on the percentage of reduction of the total heat flow compared to the growth control, and the number of colony-forming units (cfu) present in the supernatant and LVAD carrier after treatment.

**Fig 3:**
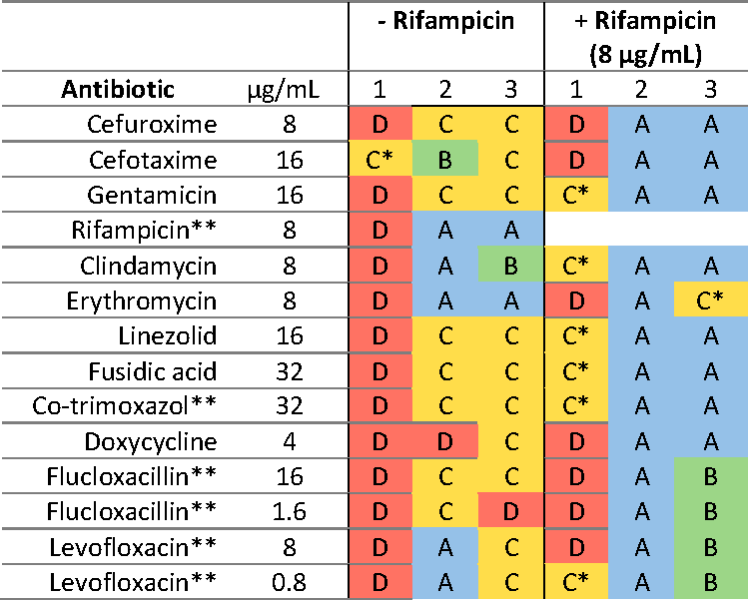
Antimicrobial efficacy of antibiotics against *S. aureus* biofilm grown on LVAD-driveline carriers in the presence or absence of rifampicin. Bacterial susceptibility is categorized from A, representing the most susceptible, to D, representing the most resistant. For some runs, no cfu in both the supernatant and carrier were detected, while the heat flow remained positive. These runs were classified one category higher than warranted by the percentage reduction in total heat flow and marked with *. Antibiotics that have been used to treat the patient are marked with **. All conditions were tested in three independent experiments.

**Fig 4:**
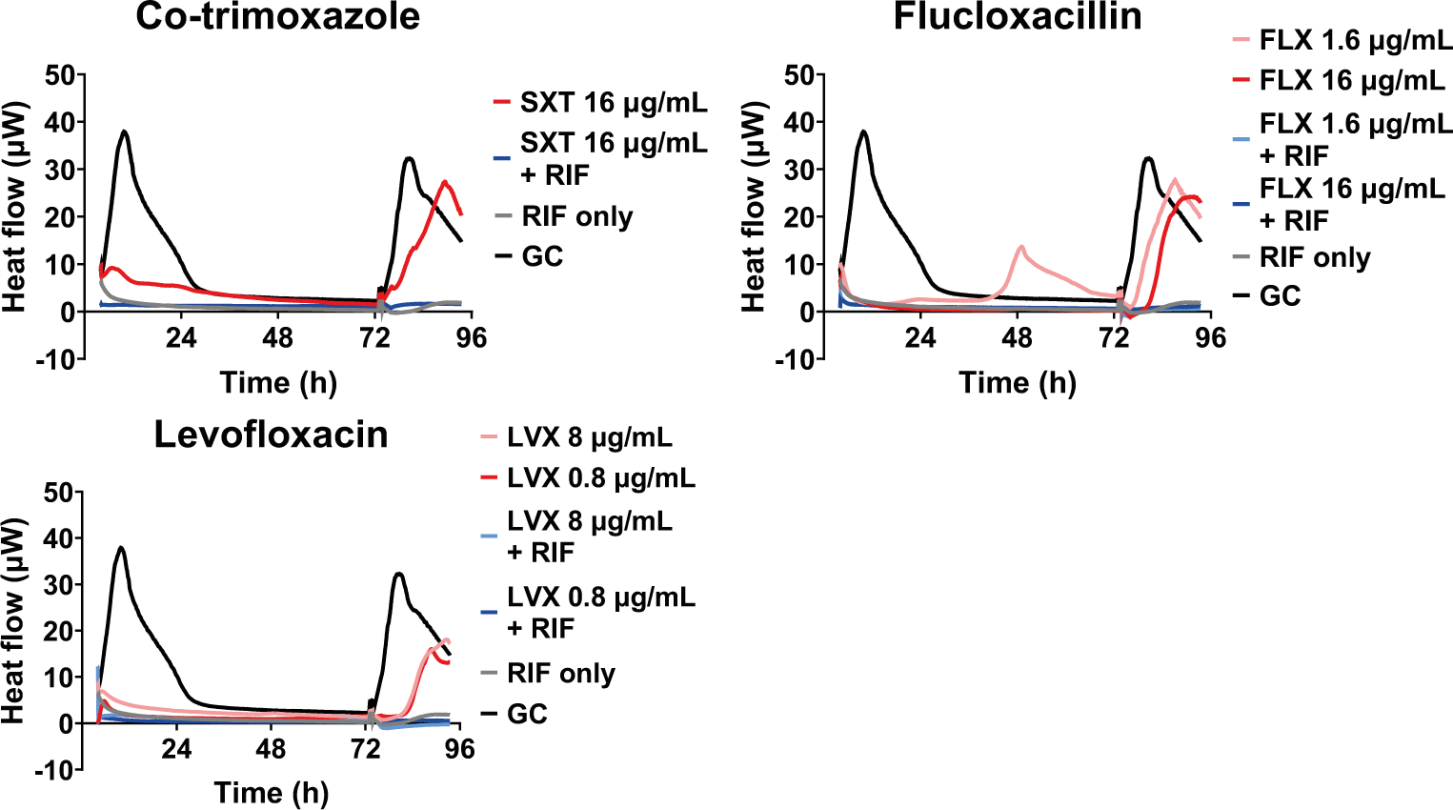
Heat curves of *S. aureus* SA-Ld biofilms treated with co-trimoxazole (SXT), flucloxacillin (FLX), or levofloxacin (LVX) individually or in combination with rifampicin (RIF, 8 µg/mL). Antibiotics were added and heat flow (µW) was measured for 72 h. A growth control (GC) with non-treated bacteria was included. After 72 h, the media was refreshed, and regrowth was monitored for an additional 24 h. The mean of three independent experiments is depicted.

Using IMC, the efficacy of various antibiotics, commonly used against *S. aureus* infections, against SA-Ld LVAD-biofilms was determined. The percentage reduction in total heat flow relative to the growth control both during treatment and after removal of the treatment (regrowth phase), together with bacterial cultures from the supernatant and LVAD-carrier, was used to categorize the antimicrobial efficacy of the treatment (Fig. 2). Although SA-Ld was susceptible to all antibiotics evaluated when grown planktonically, antibiotic susceptibility significantly decreased for its biofilms (Fig. 3). Additionally, variation between independent replicates was observed (Fig. 3; Table S2). Despite this variability, trends in antimicrobial efficacy were observed. Monotherapy with most antibiotics resulted in minimal reduction in total heat flow during treatment and regrowth, corresponding to antimicrobial efficacy levels C or D. Only treatment with erythromycin and rifampicin resulted in full bacterial clearance in most replicates, with the latter also successfully used to control the infection *in vivo*. For SXT, FLX, and LVX, which were used to treat the patient, a clear reduction in total heat flow was observed during treatment but not during regrowth (Fig. 4). These *in vitro* results correspond to the clinical treatment failure of these antibiotics when used as monotherapy for the LVAD-driveline infection *in vivo* (Fig. 1).

Combining the antibiotics with rifampicin, modeled after the *in vivo* treatment strategy, resulted in a similar susceptibility of SA-Ld to that observed with rifampicin monotherapy (Fig. 3 and 4).

### Antimicrobial efficacy of phage therapy

**Fig 5:**
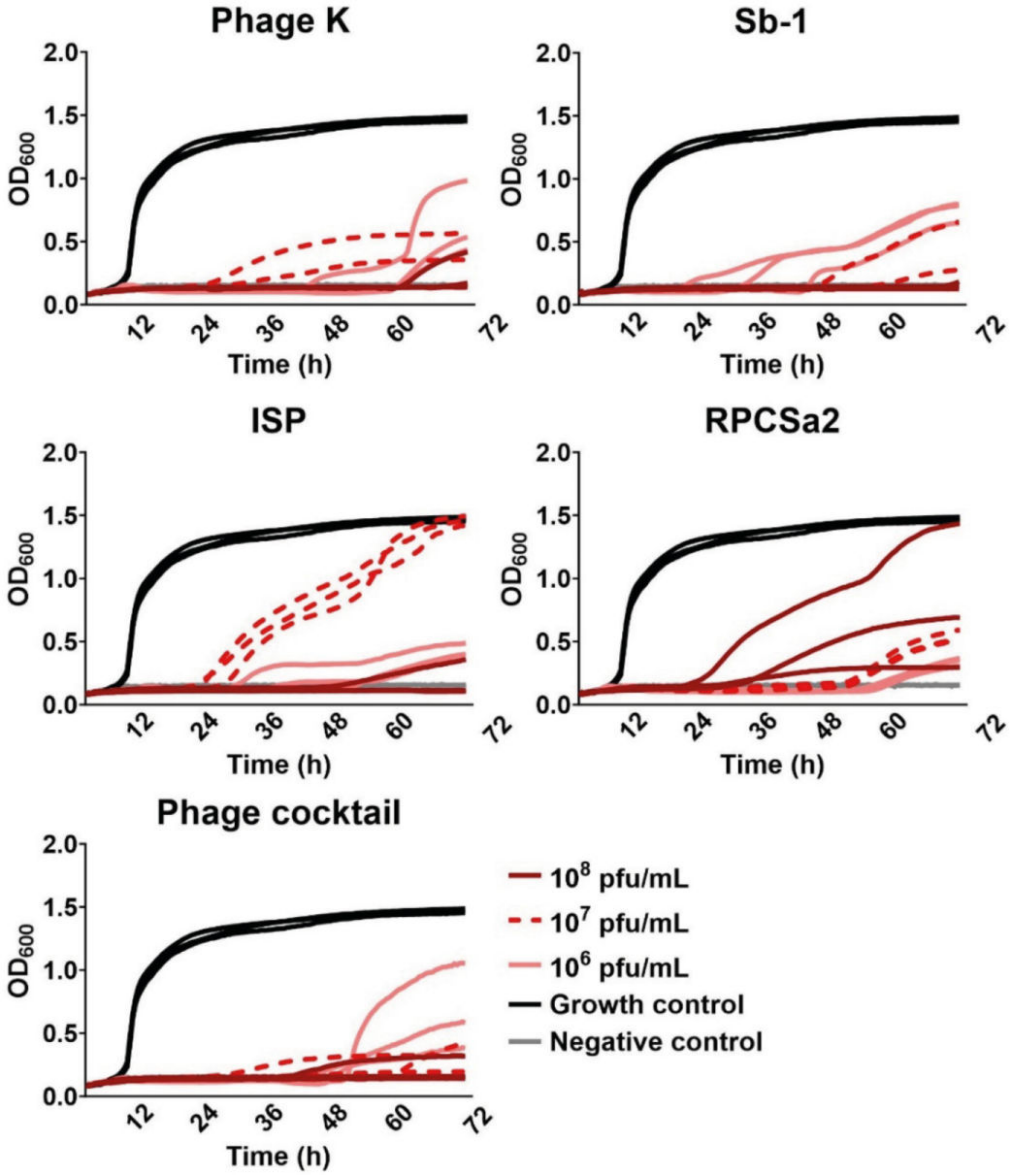
Growth curves of *S. aureus* SA-Ld treated with individual phages or a cocktail of all phages. Optical density (OD_600_) was measured for 72 h. Phages were added at concentrations of 10^8^, 10^7^, or 10^6^ pfu/mL. A medium only condition was included as a negative control (NC), and bacteria without phage treatment served as the growth control (GC). Each graph represents the results of three independent experiments.

This model was also used to investigate the potency of phage therapy. First, phage susceptibility of planktonically growing SA-Ld against four individual phages was determined using an optical density (OD) assay (Fig. 5). Each phage completely abolished bacterial growth in the first 24 h but delayed growth occurred later. At the highest phage concentration (10^8^ pfu/mL), full bacterial regrowth in all three replicates was only observed for phage RPCSa2. Combining all four phages into a phage cocktail, increased phage efficacy and delayed growth was observed only at the lowest phage concentration (10^6^ pfu/mL) after 48 h.

**Fig 6:**
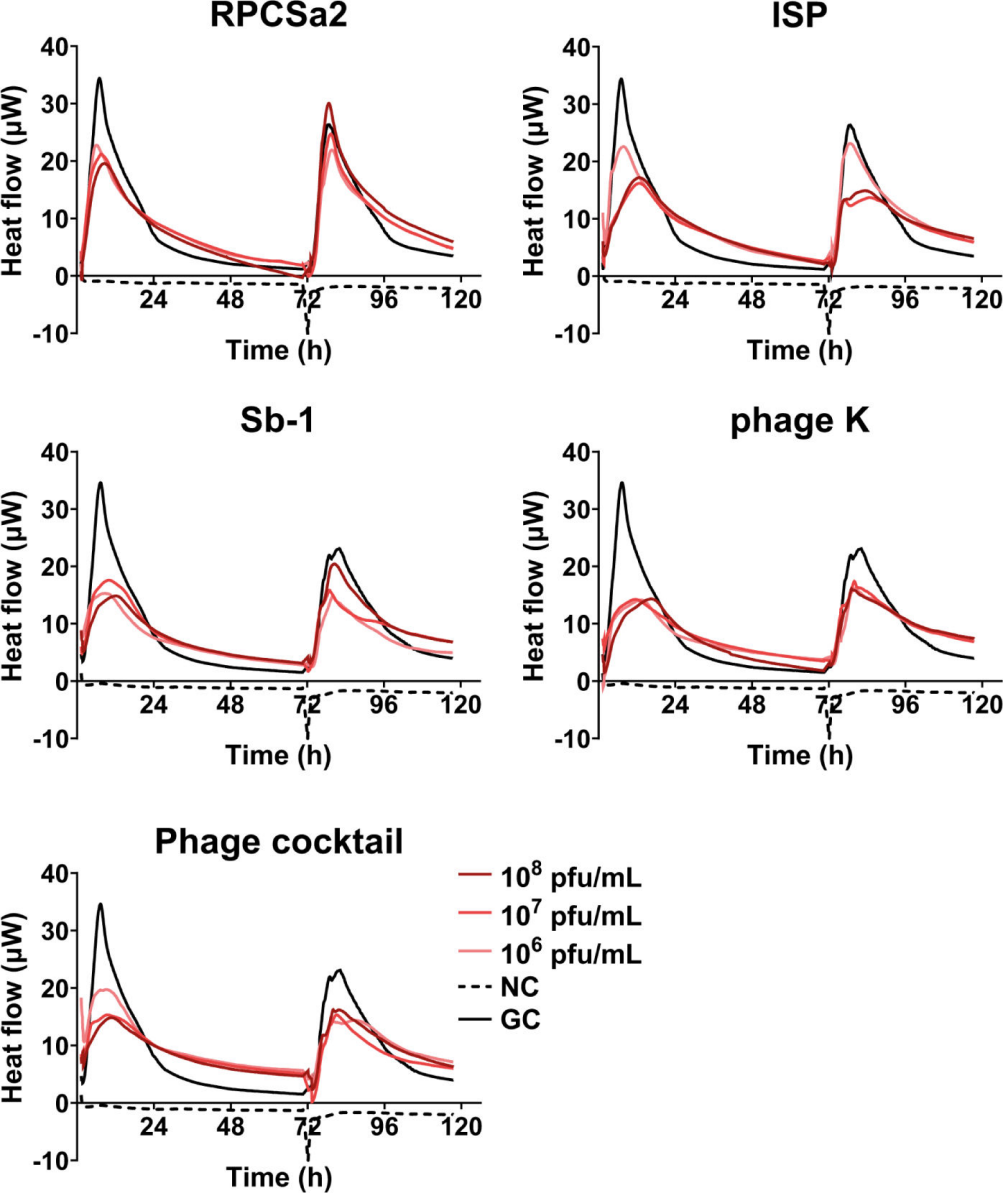
Heat curves of *S. aureus* SA-Ld biofilms treated with individual phages or a cocktail of all phages. Individual phages or phage cocktail were added at concentrations of 10^8^, 10^7^, or 10^6^ pfu/mL, and the heat flow (µW) was measured for 72 h. A negative control (NC) with medium only and a growth control (GC) with bacteria but no phage treatment was included. After 72 h, the media was refreshed, and regrowth was monitored for an additional 24 h. Three independent experiments were performed, and a representative experiment is depicted.

Next, phage efficacy was determined against the LVAD-driveline biofilms (Fig. 6). Clear heat flow was observed consistently in all conditions, during both the treatment- and regrowth phase, with less than 70% heat flow reduction (antimicrobial efficacy category D).

### Antimicrobial efficacy of flucloxacillin and phage cocktail

**Fig 7:**
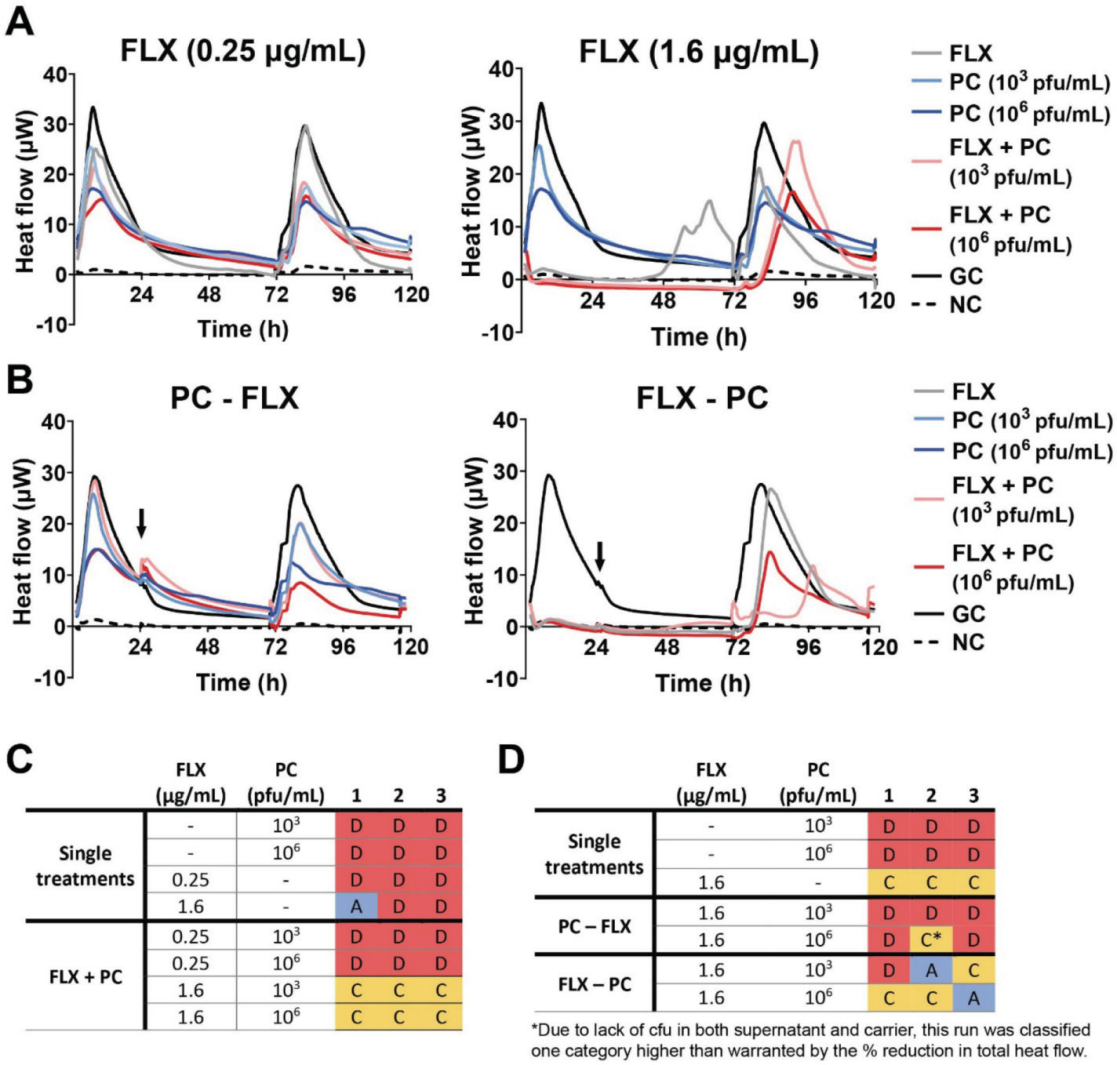
Overview of the treatment efficacy of flucloxacillin combined with the phage cocktail. (**A**) Flucloxacillin (FLX) at 0.25 or 1.6 µg/mL and the phage cocktail (PC) at 10^3^ or 10^6^ pfu/mL were added simultaneously to the *S. aureus* biofilms on LVAD-driveline. (**B**) For the staggered approach, either FLX at 1.6 µg/mL or the PC at 10^3^ or 10^6^ pfu/mL was added to the biofilm and after 24 h PC, at 10^3^ or 10^6^ pfu/ or FLX at 1.6 µg/mL, respectively, was added. The addition of the second treatment is indicated with an arrow. Heat flow was measured in µW for 72 h in total during treatment, after which the treatment was removed and regrowth was monitored for 24 h. A negative control (NC) with medium only and a growth control (GC) with bacteria but no treatment were included. Three independent experiments were performed, of which the mean is depicted. For each replicate, treatment efficacy of the (**C**) simultaneous or (**D**) staggered approach was classified into categories, with A being the most effective and D the least effective treatment.

Next, the antimicrobial efficacy of phage-antibiotic combination therapy against the LVAD-driveline biofilms was investigated. Since patients are usually treated with cocktails consisting of multiple phages (48), a combination of the four phages was used (Fig. 7A and C). FLX is the first line of treatment for methicillin-susceptible *S. aureus* LVAD infections in the Netherlands. When FLX was combined with the phage cocktail, no variation between replicates was observed (Fig. 7A; Fig. S3A); with simultaneous treatment with the phage cocktail and FLX at 0.25 µg/mL resulting in similar heat flow as the individual phage treatment (antimicrobial efficacy category D) (Fig. 7A; Fig. S3A). In contrast, combining FLX at 1.6 µg/mL, which is the maximum antibiotic concentration reached in tissue (49), with the phage cocktail resulted in antimicrobial efficacy category C (Fig. 7A and C). Compared to the single treatment with 1.6 µg/mL of FLX, the combination treatment improved antimicrobial efficacy from category D to C for two out of three replicates. However, one replicate of the FLX monotreatment achieved complete abolishment of both heat flow and cfu (category A), indicating no additional benefit for the combination treatment for that replicate.

Sequential treatment with phages and antibiotics has been shown to increase antimicrobial efficacy more than simultaneous administration; therefore, this treatment strategy was evaluated (50) (Fig. 7B and D; Fig. S4). When the phage cocktail preceded FLX treatment, most replicates showed a similar heat flow as the phage-treated biofilm during both the treatment and regrowth (Fig. 7B). Additionally, bacterial cultures were positive for both the supernatant and the carriers in all but one replicate (Fig. 7D). The antimicrobial efficacy of these replicates was categorized as level D or C* (Fig. 7B and D & Supplemental S4). In contrast, monotherapy with FLX resulted in antimicrobial efficacy level C for all replicates, indicating a lack of additive effect of this treatment strategy.

Treating with FLX followed by the phage cocktail resulted in more variable outcomes (Fig. 7B and D; Fig. S4). When FLX was combined with the phage cocktail at 10^3^ pfu/mL, it resulted in antimicrobial efficacy levels ranging from A to C. At 10^6^ pfu/mL, all runs showed over 70% reduction during treatment. In one run, full reduction in heat flow during both treatment and regrowth was observed coupled with bacterial clearance in both the supernatant and carrier (category A), while in two runs less than 70% reduction of total heat flow was observed during regrowth (category C).

PAS based on the heat flow was determined, for the treatment- and regrowth phase separately (Tables 2 and 3). Simultaneously combining FLX at 1.6 µg/mL with the phage cocktail at 10^3^ pfu/mL showed synergy in all three replicates during the treatment phase (Table 2). During the regrowth phase, PAS was observed in two out of three replicates for FLX (0.25 µg/mL) combined with phage cocktail (10^6^ pfu/mL). Greater variation in PAS was observed with the sequential approach compared to simultaneous administration, with none of the sequential conditions achieving PAS in all three replicates (Table 3).

**TABLE 2:** Calculation of phage-antibiotic synergy during and after simultaneous treatment with flucloxacillin and the phage cocktail^*a*^

	FLX (µg/mL)	PC (pfu/mL)	Replicate 1	Replicate 2	Replicate 3
Treatment phase	0.25	10^3^	0.69	0.60	**1.10**
	0.25	10^6^	0.84	0.92	0.91
	1.6	10^3^	**4.08**	**>10**	**>10**
	1.6	10^6^	0	0	0
Regrowth phase	0.25	10^3^	0.66	0.65	**1.26**
	0.25	10^6^	**1.05**	**1.08**	0.97
	1.6	10^3^	0.01	0.55	0.91
	1.6	10^6^	0.01	1.00	**1.03**

^*a*^ Values >1 represent phage-antibiotic synergism and are marked in bold. All conditions were tested in three independent experiments.

**TABLE 3:** Calculation of phage-antibiotic synergy during and after staggered treatment with flucloxacillin and the phage cocktail^*a*^

	FLX (µg/mL)	PC (pfu/mL)	Replicate 1	Replicate 2	Replicate 3
Treatment phase PC – FLX	1.6	10^3^	0.02	0.01	0.03
	1.6	10^6^	0.02	0.02	0.05
Regrowth phase PC – FLX	1.6	10^3^	**1.29**	0.73	0.79
	1.6	10^6^	0.91	**>10**	**1.41**
Treatment phase FLX – PC	1.6	10^3^	0.09	0.32	**4.58**
	1.6	10^6^	0	0	**2.37**
Regrowth phase FLX – PC	1.6	10^3^	**2.21**	**>10**	0.78
	1.6	10^6^	0.68	0.88	**>10**

^*a*^ Values >1 indicate phage-antibiotic synergism and are marked in bold. All conditions were tested in three independent experiments.

### Antimicrobial efficacy of flucloxacillin, rifampicin, and phage cocktail

**Fig 8:**
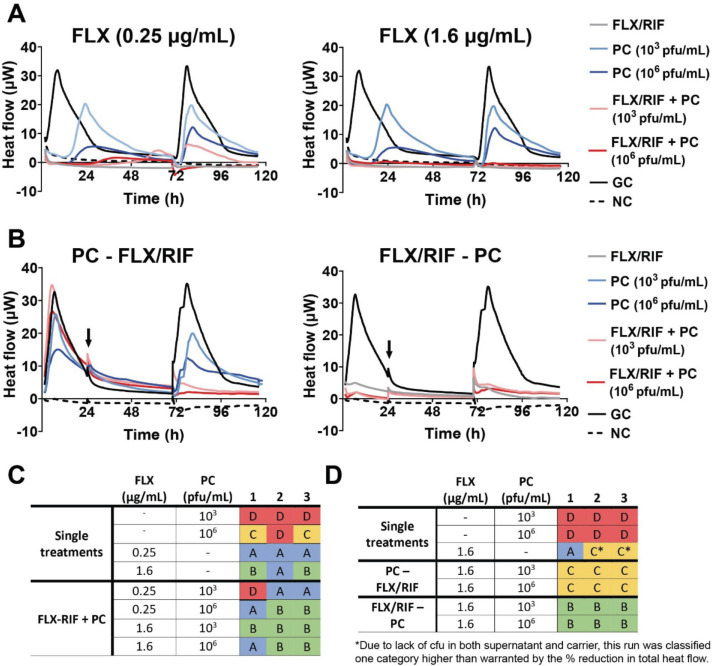
Overview of the treatment efficacy of flucloxacillin with rifampicin combined with the phage cocktail. Flucloxacillin (FLX) at 0.25 or 1.6 µg/mL was combined with 8 µg/mL rifampicin (RIF). These antibiotics were combined with the phage cocktail (PC) at 10^3^ or 10^6^ pfu/mL and were then (A) added to the *S. aureus* biofilms. (**B**) For the staggered approach, RIF was combined with FLX at 1.6 µg/mL and added to the biofilms 24 h before or after the PC at 10^3^ or 10^6^ pfu/mL. The addition of the second treatment is indicated with an arrow. Heat flow was measured in µW for 72 h during treatment and 24 h after removal of the treatment. A negative control (NC) with medium only and a growth control (GC) (with bacteria but no treatment) were included. Three independent experiments were performed of which the mean is depicted. For each replicate, treatment efficacy of the (**C**) simultaneous or (**D**) staggered approach was classified into categories with A being the most effective and D the least effective treatment in three independent experiments.

Since combining antibiotics with rifampicin was a successful strategy for treatment of the LVAD-driveline infection *in vivo*, antimicrobial efficacy of the phage cocktail together with both FLX and rifampicin was evaluated (Fig. 8). Adding the phage cocktail to the biofilm simultaneously with both antibiotics resulted in high antimicrobial efficacy; level A or B for most replicates, which was similar to the antibiotic mono-treatment (Fig. 8A C; Fig. S3B).

Next, the effect of a sequential treatment strategy was evaluated (Fig. 8B and D). Variation between replicates was observed for the antibiotic monotherapy (Fig. 8D; Fig. S5 and Table S4). With one replicate showing both low cfu counts and a high reduction of heat flow (category A), while the two other replicates did show a high (>70%) reduction in total heat flow during regrowth, but not during treatment (category C*, Fig. 8B and D). When administration of the phage cocktail preceded FLX and rifampicin, the reduction in heat flow during the regrowth phase was less than 70% and full bacterial growth was observed in all replicates (category C), which was less efficient than monotherapy with the antibiotics (Fig. 8D). Administering both FLX and rifampicin prior to the phage cocktail resulted in over 70% reduction of the total heat flow during both treatment and regrowth phases. This reduction was similar to that observed for the monotherapy with the antibiotics (Fig. 8B; Fig. S5). Positive bacterial cultures were observed for all replicates resulting in category B for all replicates (Fig. 8C and D).

Due to the lack of total heat flow during both treatment and regrowth with FLX and rifampicin, PAS could not be calculated for these conditions.

## DISCUSSION

LVAD-related infections, especially of the driveline, occur often and can have detrimental consequences (51, 52). Antibiotic resistance and biofilm formation complicate treatment for LVAD infections (52). Here, the efficacy of antibiotic monotherapy, and in combination with rifampicin, against a LVAD-driveline infection caused by *S. aureus* was evaluated. In addition, the potency of phages and phage-antibiotic combination treatment against LVAD-driveline biofilms was determined. For this purpose, a novel assay using isothermal microcalorimetry (IMC) was developed. In contrast to standard biofilm susceptibility assays, IMC is less labor intensive, faster, and more reproducible (8). Additionally, this is the first time IMC has been applied to grow *S. aureus* in conditions particular for LVAD-driveline infections; growing biofilms on LVAD-driveline carriers in cell culture medium supplied with serum.

Using this assay reduced antibiotic susceptibility of *S. aureus* biofilms, grown on LVAD-driveline, was observed. This finding confirms previous studies showing antibiotic susceptibility of biofilms can decrease up to 1,000-fold compared to their planktonic counterparts (8, 9). Whereas most antibiotics showed decreased efficacy against the biofilms, two antibiotics (rifampicin an erythromycin) completely abolished bacterial growth in two out of three replicates. Of these, rifampicin has also been used to treat the patient and eventually resolved the LVAD-driveline infection. Several clinical studies have demonstrated anti-biofilm activity of rifampicin, which is potentially linked to its ability to kill persister cells (10, 53). Although combination therapy with rifampicin did not drastically increase antimicrobial efficacy over monotherapy, it remains highly recommended due to the high risk of rifampicin resistance when using rifampicin monotherapy (10, 53).

Similar to antibiotics, phage monotherapy showed high activity against planktonic bacteria but limited efficacy against biofilms, consistent with prior studies reporting biofilm reduction, not eradication, by phage K and phage Sb-1 (16, 20, 21, 41–43, 45). Antimicrobial efficacy of combined treatment with the phage cocktail and FLX was dependent on the antibiotic concentration, while phage concentrations did not seem to influence treatment efficacy. Next to antibiotic concentration, treatment order also influenced antimicrobial efficacy. Previous studies have shown enhanced antimicrobial activity when phage administration preceded antibiotic treatment (16, 54). However, this strategy does not align with current clinical practice in Western-Europe, where phage therapy is applied only as a last resort after antibiotic treatment has failed (55, 56). In contrast, this study did not observe a benefit of phage application prior to antibiotics. On the contrary, this treatment strategy even increased bacterial growth compared to antibiotic monotherapy. This reduction in antimicrobial efficacy may be due to an initial reduction in bacterial cells by FLX, resulting in decreased phage production and phage-mediated lysis (57). Administering FLX prior to the phages resulted either in similar antimicrobial efficacy as monotherapy or in full bacterial clearance, suggesting this sequential treatment strategy might be the more beneficial. Altogether, these findings suggest that not only the choice of antibiotics and phages, but also antibiotic concentration and treatment order can significantly influence antimicrobial efficacy of combination treatment.

Throughout this study, discrepancies between independent experimental replicates were observed. Although the coefficient of variance (CoV) exceeded 15% in some cases, indicating limitations of the assay, it remained below this threshold for the majority of phage-antibiotic combinations and growth controls, particularly during the regrowth phase. While the assay should be further optimized to reduce inter-experimental variation, these findings also suggest the presence of biological variability. While this limits the ability to draw clear conclusions on treatment efficacy, it also highlights the difficulties encountered when examining and treating biofilms *in vivo*. As mentioned, this assay specifically monitors treatment efficacy on biofilms, which are known to display heterogeneity (8–13). Even biofilms grown under the same conditions can vary heavily from each other, with both adaptive and phenotypical variation between biofilms influencing treatment efficacy of both antibiotics and phages (13, 21, 58). One cause of biofilm heterogeneity can be the emergence of subpopulations within the biofilm, such as persister cells. These bacterial cells exhibit low or no metabolic activity, making them less susceptible to both antibiotics and phages (21, 58, 59). Notably, IMC only detects heat produced by metabolically active bacteria, meaning variation in the number of persister cells within a biofilm can cause variation in antimicrobial efficacy of both antibiotics and phages. Other sources of biofilm heterogeneity include biofilm architecture, cellular arrangement, and surface morphology. These factors likely also introduce biofilm variability *in vivo* (24, 60, 61). Another cause for the variability observed in this study may be the use of physiologically relevant culture conditions. Contrarily, most studies use bacterial cell culture medium to study biofilms, such as Tryptic Soy Broth and Brain Heart Infusion, often supplied with 1% glucose (27–29, 34, 45). While these media minimize biofilm heterogeneity and improve reproducibility (9, 27–29, 34, 45), they do not resemble the conditions encountered *in vivo*. Nevertheless, these conditions do influence phage efficacy (24). For example, the presence of serum can inhibit phage activity (47, 62–64). Additionally, variations in glucose levels can influence persister cell formation (12, 58). Finally, glycosylation of teichoic acid, a *S. aureus* phage receptor, is affected by environmental conditions, further influencing phage efficacy (65, 66). Future research into the biofilm composition of *S. aureus,* particularly under conditions relevant to LVAD infections, is needed to provide more insight into the antibiotic and phage susceptibility of these biofilms.

Although the variability in this study hinders the ability to draw clear conclusions, it may provide a more accurate insight in the efficacy of both phages and antibiotics in conditions encountered *in vivo* during a LVAD-infection. Furthermore, this study was conducted using a *S. aureus* strain which was isolated from a LVAD-driveline infection, from a patient with a well-documented treatment history. This allowed for comparison of the *in vitro* activity of the antibiotics to the efficacy of the *in vivo* antibiotic treatment, increasing the clinical relevance of this study. However, the use of this single strain limits the breadth of this research, so additional bacterial strains (and phages) should be included in future studies to further validate this assay and to better extrapolate the data to the clinic. Additionally, the combination of phages with a broader range of antibiotics using additional concentrations of both agents should be evaluated. Overcoming these current limitations will be essential to improve this assay and use it to provide guidance for antibiotic treatment of LVAD-driveline infections. Moreover, the assay could be used to improve phage selection for therapy in the future. Currently, PAS and biofilm formation are hardly considered during phage selection. For example, in the 20 published case reports on phage therapy for LVAD infections, phages were selected based on their efficacy against planktonic bacteria, rather than on biofilms. Furthermore, in all cases, phage therapy was combined with antibiotic treatment (4, 26–34), while only one study (three patients) evaluated PAS before treatment (32). Taken together, only 55% (11/20) of these cases resulted in a successful treatment of the infection. Considering both biofilm formation and PAS, prior to phage therapy is paramount to achieve more consistent outcomes in future phage therapy (6, 67).

Altogether, this study emphasizes the complexity of LVAD-driveline infections as well as the variability of biofilms, especially when grown in physiologically relevant conditions. Despite its limitations, this study may serve as a stepping stone for the evaluation of biofilm heterogeneity and help guide antibiotic and phage treatment strategies for LVAD infections. Additionally, this model could be used for *in vitro* phage selection prior to therapy, to ensure the use of phages with anti-biofilm activity and PAS. Collectively, these efforts may improve treatment strategies for difficult-to-treat *S. aureus* LVAD-driveline infections.

## MATERIALS AND METHODS

### Bacterial isolates and bacteriophages

#### Bacterial isolates

A 59-year-old patient with ischemic cardiomyopathy, for which he received a left ventricular assist device in June 2016, was admitted to the Erasmus University Medical Center (Rotterdam, The Netherlands) at the end of May 2018 (Fig. 1). The patient exhibited redness and purulent discharge along the driveline exit and was diagnosed with a deep driveline infection. The causative pathogen was isolated on 27 July 2018 and identified as methicillin-susceptible *S. aureus* strain SA-Ld (ST5).

*S. aureus* strain R5 is highly sensitive to phages and has historically been used for the propagation of phages for phage typing (47, 68). Therefore, this strain was used for phage production. Strains were stored in glycerol at −80°C and sub-cultured overnight on Tryptic Soy Agar II plates with 5% sheep blood (TSA II) (BD, Franklin Lakes, NJ, USA) at 37°C prior to experiments.

#### Bacteriophages

Bacteriophage K is a polyvalent phage first described in 1935, under the name Au2. Phage K is known for its ability to reduce and prevent biofilm formation (41–44). Phage RPCSa2 was isolated from a phage cocktail produced in Russia and is genetically similar to phage K. This phage has a broad host range infecting both methicillin susceptible- and methicillin-resistant *S. aureus* (MRSA) strains (47). Bacteriophage ISP was kindly provided by dr. Rob Lavigne from LU Leuven (Belgium) and was originally isolated in the 1920s by the Eliava Institute in Georgia from an unknown source. In addition, phage ISP has previously been used to treat patients with burn wound infections (69, 70). Phage Sb-1 was isolated in 1977 from a phage cocktail produced by the Eliava Institute (46). This phage has been used to treat *S. aureus* LVAD infections and is able to degrade biofilms (16, 17, 21, 45, 46).

### Bacteriophage production

*S. aureus* strain R5 was grown in 100 mL tryptic soy broth (TSB) at 37°C. When the bacteria reached the exponential growth phase (OD_600_ = 0.5 ± 0.2), 20 µL phage was added. The suspension of bacteria and phages was incubated overnight at 37°C shaking at 150 rpm. The next day, the phage lysate was centrifuged at 4,000 × *g* for 40 min at 4°C. The supernatant was recovered and filtered using a 0.22 µm Whatman puradisc filter (Merck KGaA, Darmstadt, Germany). The suspension was further purified and concentrated using Zeba Spin Desalting columns with a 40 kDa cut off (ThermoFisher Scientific, Waltham, USA) and the PEG virus precipitation kit (Abcam, Cambridge, UK), both used according to the manufacturer’s protocol. The precipitated virus was resuspended in SM buffer (100 mM NaCl, 8 mM MgSO_4_.7H_2_O, and 1 M Tris-CI, pH 7.5). Phage titers were determined through a spot test as described previously (47). In short, a single colony of R5 was incubated in Luria-Bertani (LB) broth (Merck KGaA, Darmstadt, Germany) at 37°C and grown until the exponential phase (OD_600_ = 0.5 ± 0.2). Two hundred microliters of bacteria was added to 4 mL 0.35% LB agar (containing 1 M CaCl_2_ and 1 M MgSO_4_) and poured onto a 1.4% LB agar plate. Ten-fold dilutions of each phage were prepared in phosphate buffered saline (PBS). When the 0.35% LB agar solidified, 10 µl of each phage dilution was pipetted onto the plate. Plates were incubated overnight at 37°C, and plaque-forming units (pfu) were determined the next day.

### Susceptibility testing of planktonic *S. aureus*

#### Antibiotic susceptibility

Once isolated from the patient, antibiotic susceptibility of the *S. aureus* strain SA-Ld to various antibiotics (Table S1) was determined using the VITEK 2 system (bioMérieux Benelux B. V, Zaltbommel, the Netherlands), according to manufacturer protocols, and cefoxitin disk diffusion (Liofilchem, Roseto degli Abruzzi, Italy). Results were interpreted according to EUCAST clinical breakpoints.

#### Bacteriophage susceptibility

An 0.5 McFarland solution of SA-Ld was prepared from an overnight culture on a TSA II plate. This solution was diluted in Iscove’s modified Dulbecco’s medium (IMDM) (ThermoFisher Scientific, Waltham, USA) to a final concentration of 10^5^ colony-forming units (cfu)/mL. Single bacteriophages were diluted in PBS to a final concentration of 10^8^, 10^7^, and 10^6^ pfu/mL. To prepare the phage cocktail, all four single phages were combined in equal parts to a combined concentration of 2 × 10^9^ pfu/mL, and 10-fold dilutions were prepared. Ten microliters of either the single phage or phage cocktail dilutions was added to 190 µL of the bacterial suspension in a round bottom 96-wells plate (Greiner, Kremsmüster, Austria). The plate was incubated at 37°C, and optical density (OD_600_) was measured every 30 min for 72 h using the Agilent BioTek Epoch 2 microplate spectrophotometer (ThermoFisher Scientific, Waltham, USA). All experiments were performed in triplicate, and OD_600_ growth curves were visualized using Graphpad Prism (v9).

### Susceptibility testing of *S. aureus* biofilms

#### Biofilm establishment

An LVAD-driveline was removed from a patient and was scrubbed using NaCl; for further cleaning, the driveline was incubated overnight in 70% EtOH and then autoclaved at 120°C. The cleaned LVAD driveline was cut into rings 4 mm in diameter, which were halved. These carriers were incubated with *S. aureus* SA-Ld for 3 days in IMDM with 10% fetal calf serum (IMDM/10% FCS) at 37°C while shaking at 175 rpm. To remove planktonic bacterial cells, the carriers were washed daily with IMDM/10% FCS. After 3 days, the carriers were transferred to non-activated CalWel sterile inserts (SymCel, Solna, Sweden), containing 200 µL IMDM/10% FCS. One carrier was placed inside each insert, which was then positioned in a titanium cup (SymCel, Solna, Sweden). Heat flow was measured for 24 h at 37°C using the CalScreener (SymCel, Solna, Sweden) to monitor metabolic heat flow of the biofilms. After 24 h, the heat flow was evaluated; if no major variation between carriers was observed, the experiment was continued. Carriers were washed once with IMDM/10% FCS. Subsequently, carriers were transferred into clean CalWel inserts containing the treatment.

#### Treatment

Each antibiotic was diluted in IMDM/10% FCS to twice its peak concentration achievable *in vivo* in serum or tissue (Table S1). Additionally, the antibiotics were combined with rifampicin (8 µg/mL). A total of 100 µL of the antibiotic dilution was added to the carriers along with either 100 µL of IMDM/10% FCS or 100 µL of the rifampicin dilution. For bacteriophage treatment, individual phages were diluted in PBS; for the phage cocktail, all individual phages were combined in equal parts. Then, 190 µL of IMDM/10% FCS was added to the carriers in the CalWel inserts. Ten microliters of phage dilution was added, resulting in final phage concentrations of 10^8^, 10^7^, and 10^6^ pfu/mL. For all experiments, untreated carriers were included as growth control, and a medium only condition as negative control. The inserts were placed back into the titanium vials and measurements resumed at 37°C for 72 h.

#### Determination of regrowth

After 72 h, measurements were interrupted and supernatant containing the treatment was removed. The carriers were washed once with NaCl and were transferred to clean CalWel inserts containing 200 µL IMDM/10% FCS. Measurements were continued at 37°C for 48 h.

#### Culturing of supernatant and carriers

After 48 h, IMC measurements were discontinued, and 50 µL of the supernatant from each CalWel insert was plated on TSA II plates. In addition, the carriers were washed twice with NaCl and swabbed across a TSA II plate while exerting pressure (Fig. S1). All plates were incubated overnight at 37°C after which the number of residual cfu was determined. All experiments were performed in triplicate.

#### Determination of total heat flow

Similar to previous work (71), a smoothed spline was fit to the time series data, with all negative values set to zero, using either data of the treatment- or regrowth period. Integration over this fitted spline gave the area under the curve values (AUC) or total heat flow in that period.

### Assessment of phage-antibiotic synergy

#### Simultaneous treatment

Biofilms were established for IMC as described above. The bacteriophage cocktail was prepared in PBS as described above to 2 × 10^5^ and 2 × 10^7^ pfu/mL. Flucloxacillin (FLX) was diluted in IMDM/10% FCS to 5 and 32 µg/mL. Carriers were submerged in 180 µL IMDM/10% FCS to which 10 µL of the FLX dilutions (final concentration 0.25 and 1.6 µg/mL) and 10 µL of the phage cocktail were added (final concentration 10^3^ and 10^6^ pfu/mL). For treatment with antibiotic alone, 10 µL of PBS was added instead of the phage cocktail. For the treatment with only phages, 10 µL of IMDM/10% FCS was added instead of FLX. To determine antimicrobial efficacy of FLX combined with rifampicin and the phage cocktail, rifampicin was diluted to 160 µg/mL in IMDM/10% PBS. The carriers were submerged in 170 µL of IMDM/10% FCS to which 10 µL of FLX with 10 µL rifampicin (final concentration 8 µg/mL) and 10 µL of phage cocktail were added. For individual antibiotic treatment, 10 µL of PBS was added instead of the phage cocktail. For treatment with phage alone, 20 µL of IMDM/10% FCS was added instead of FLX and rifampicin. For all experiments, untreated carriers were included as growth control, and a medium only condition as the negative control. Antimicrobial efficacy, regrowth, and CFU were determined as described above. All experiments were performed in triplicate, IMC data were analyzed with CalView (SymCel, Solna, Sweden), and heat curves were visualized using Graphpad Prism (v9).

#### Sequential treatment

Antibiotic and phage dilutions were prepared as described above. After the first 24 h of biofilm establishment, 10 µL of the phage cocktail or FLX (with or without rifampicin) was added to each carrier. Twenty-four hours later, the measurement was disrupted to add 10 µL of the other treatment. Measurements were continued for another 48 h, after which antimicrobial efficacy, regrowth, and CFU were determined as described above.

#### Determination of synergy

A combination of phages and antibiotics was deemed synergetic according to the definition of Chaudhry et al. (54) (54). They describe synergy as the fraction of surviving cells after antibiotic treatment (Sa) or phage treatment (Sb), divided by the cell density of the growth control (C). When the cell survival of antibiotic treatment multiplied by survival after phage treatment is greater than the cell survival after combined treatment (Sab), the combination is considered synergistic (54, 72).

1. Synergy = (Sa/C) * (Sb/C) > Sab/C
2. Synergy = (Sa/C) * (Sb/C)/(Sab/C) >1

The total heat flow of each condition was used to assess the presence of synergism during both treatment and regrowth period across various phage-antibiotic combinations.

## SUPPLEMENTAL MATERIAL

Supplemental Material jcm.00272-25-s0001.docx Tables S1 to S4; Fig. S1 to S5.

ASM does not own the copyrights to Supplemental Material that may be linked to, or accessed through, an article. The authors have granted ASM a non-exclusive, world-wide license to publish the Supplemental Material files. Please contact the corresponding author directly for reuse.
