# Supplementary material for: In vitro activity of antibiotic monotherapy and combination therapy with bacteriophages against Staphylococcus aureus LVAD-driveline infections
Source: J Clin Microbiol. 2025 Oct 9;63(11):e00272-25. doi: 10.1128/jcm.00272-25 (PMC12607560; doi:10.1128/jcm.00272-25)
Supplement: Supplemental Material — Tables S1 to S4; Fig. S1 to S5. [file jcm.00272-25-s0001.docx]

**Supplemental material**

**Supplemental Table S1: Overview of antibiotics investigated for efficacy on *S. aureus* biofilms.**

| **Antibiotic** | **Manufacturer** | **Peak serum concentration (µg/mL)** | **Peak tissue concentration (µg/mL)** |
| --- | --- | --- | --- |
| Flucloxacillin* | Aurobindo Pharma B.V.  (Baarn. The Netherlands) | 16 | 1.6 |
| Cefuroxime | Sigma-Aldrich  (Missouri. United States) | 8 |  |
| Gentamicin | Erasmus MC pharmacy  (Rotterdam. The Netherlands) | 16 |  |
| Cotrimoxazole* | Roche Nederland B.V.  (Woerden. The Netherlands) | 32 |  |
| Levofloxacin* | Sigma  (Missouri. United States) | 8 | 0.8 |
| Erythromycin | Sigma  (Missouri. United States) | 8 |  |
| Clindamycin | Sigma  (Missouri. United States) | 8 |  |
| Rifampicin* | Sigma  (Missouri. United States) | 8 |  |
| Fusidic acid | Sigma  (Missouri. United States) | 32 |  |
| Linezolid | Sigma  (Missouri. United States) | 16 |  |
| Doxycycline | Sigma  (Missouri. United States) | 4 |  |
| Cefotaxime | Duchefa Farma B.V.  (Haarlem. The Netherlands) | 16 |  |

***Antibiotic used to treat the patient.


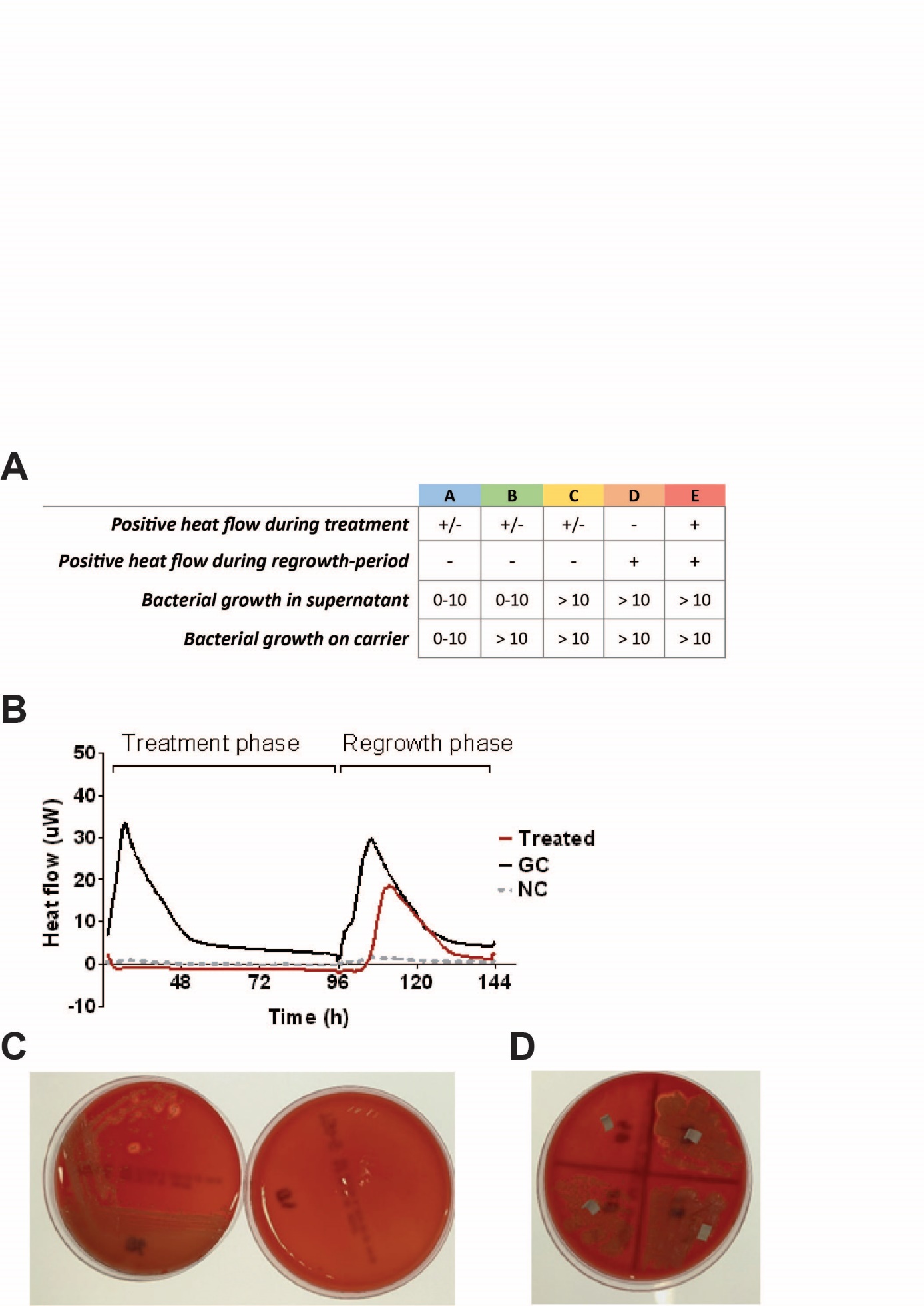

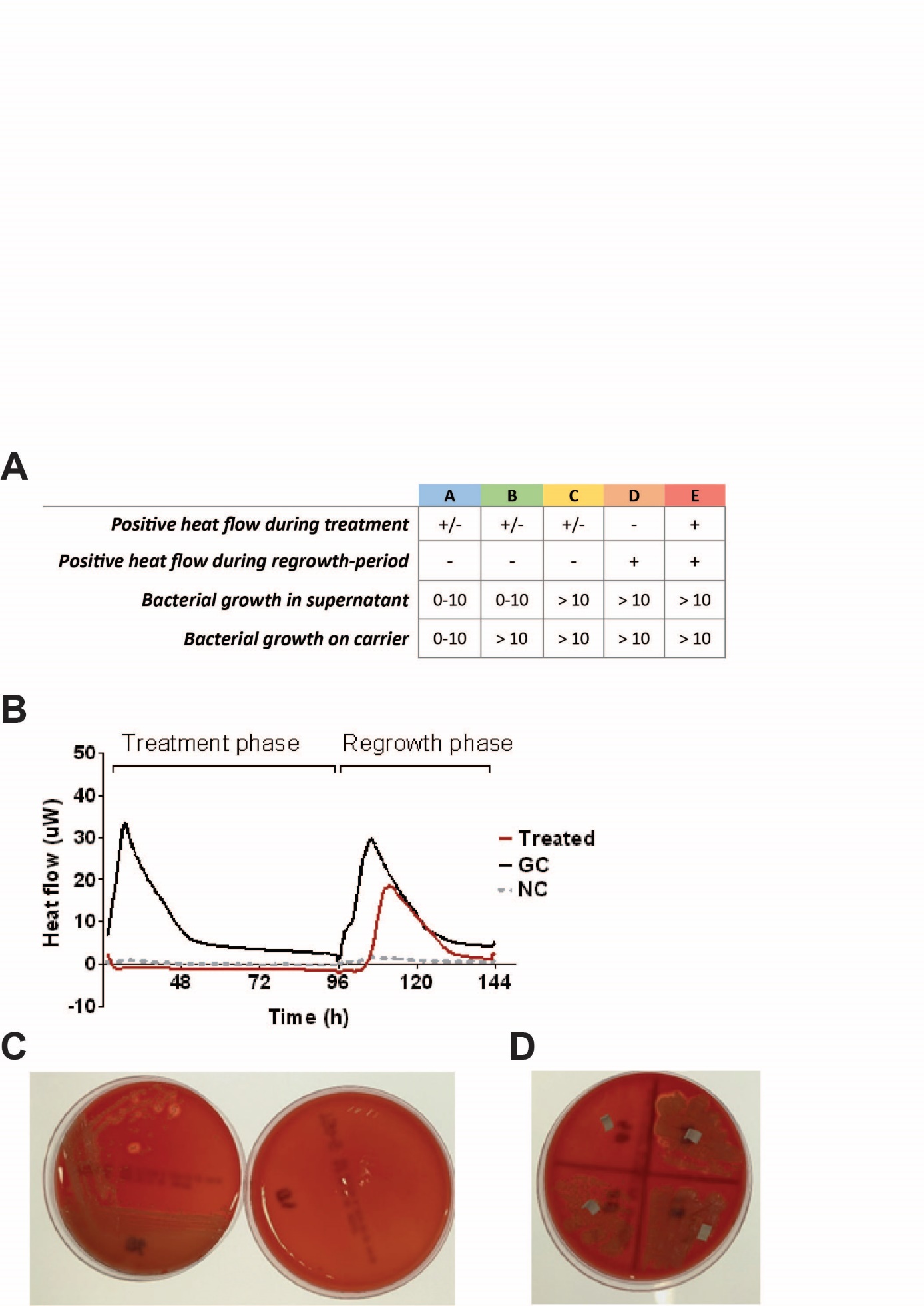


**Supplemental figure S1: Bacterial cultured used for antimicrobial efficacy categorization.** After each experiment both the supernatant and LVAD-carriers were cultured to determine bacterial growth. Example of a positive (left) and negative (middle) supernatant cultures are depicted. LVAD carriers (white) were swapped across a section of the plate (right) and then cultured. a negative carrier is depicted in the top left section while the other section exhibit bacterial growth.

**
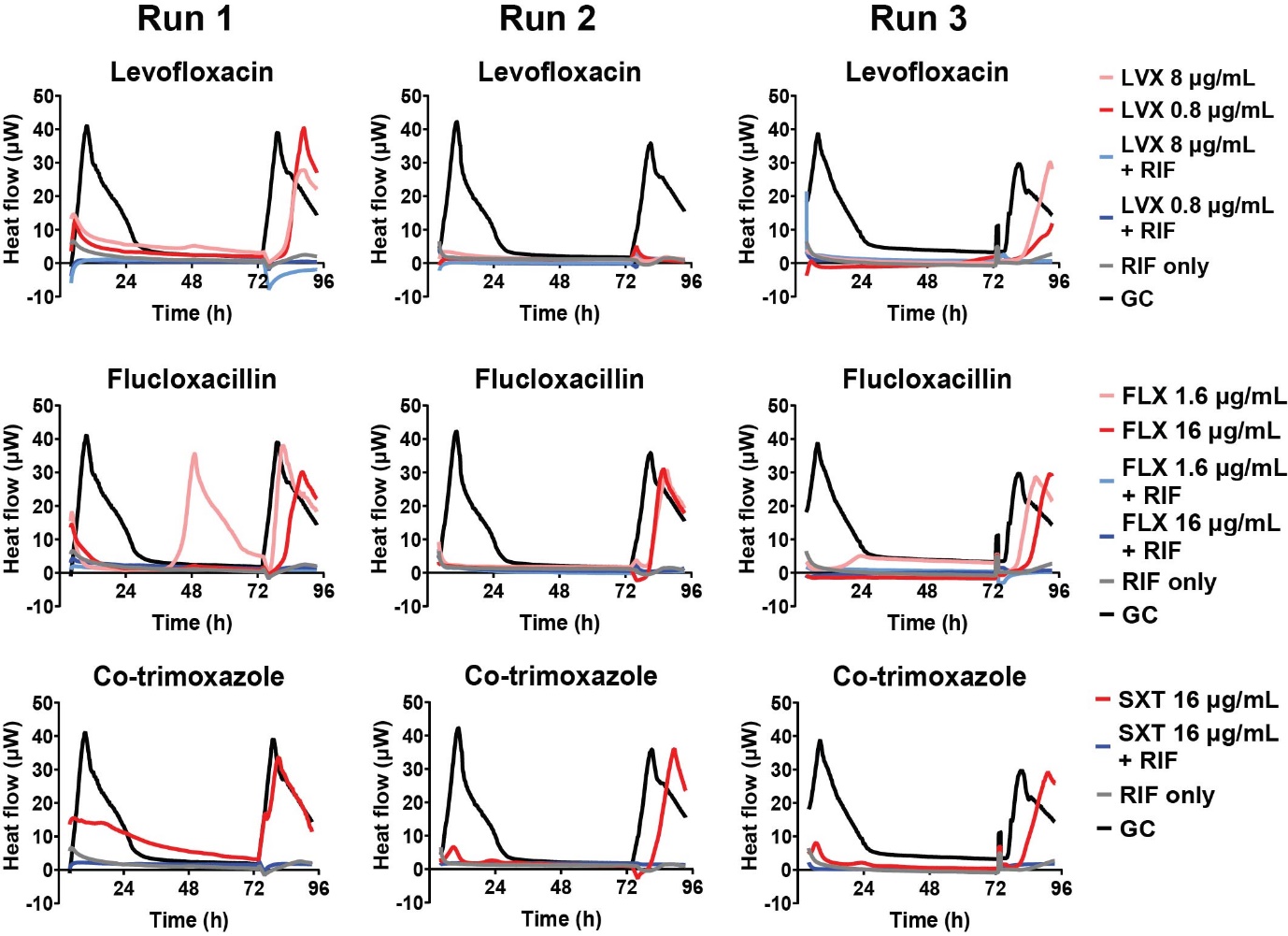
**

**Supplemental figure S2: Heat** **flow curves of *S. aureus* biofilms treated with individual antibiotics or in combination with rifampicin.** Levofloxacin (LVX). flucloxacillin (FLX) or co-trimoxazole (SXT) were added either individually or in combination with rifampicin (RIF, 8 µg/mL) to the *S aureus* biofilm. Heat flow (µW) was measured in µW for 72 hours during treatment. After 72 hours the treatment was removed and regrowth was monitored for 24 hours. A medium only condition was included as negative control and an untreated condition as growth control. Three independent experiments are shown (Run 1-3).

**
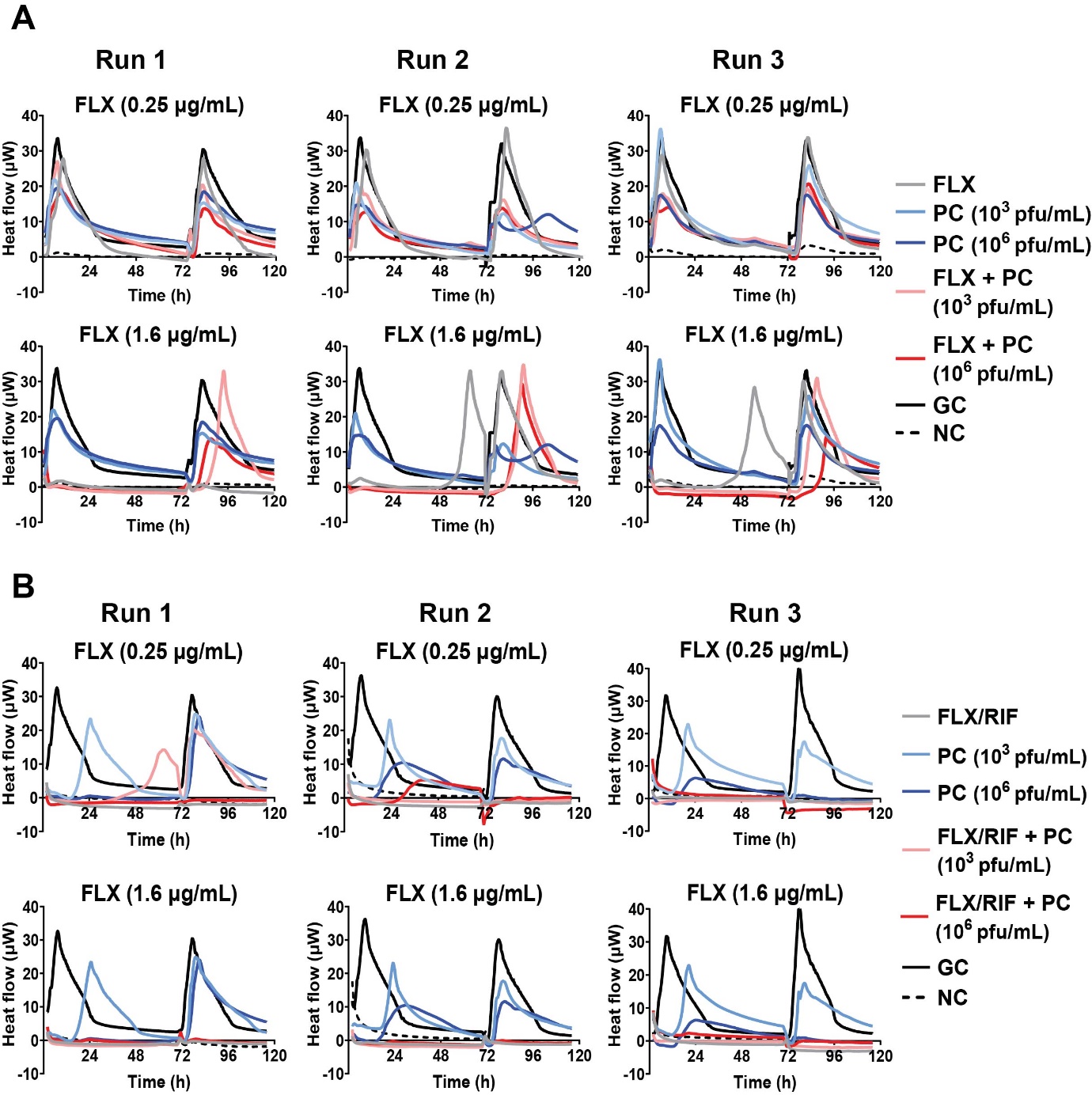
**

**Supplemental Figure S3**: **Heat** **flow curves of *S. aureus* biofilms treated with the phage cocktail and antibiotics.** A) Flucloxacillin (FLX) at 0.25 or 1.6 µg/mL was simultaneously added to the *S. aureus* biofilms with the phage cocktail (PC) at 10^3^ or 10^6^ pfu/mL. B) Flucloxacillin was combined with both the phage cocktail and 8 µg/mL rifampicin (RIF). Heat flow of the LVAD-driveline biofilms was measured in µW for 72 hours during treatment. After 72 hours the treatment was removed and regrowth was monitored for 24 hours. A medium only condition was included as negative control and an untreated condition as growth control. Three independent experiments are shown (Run 1-3).


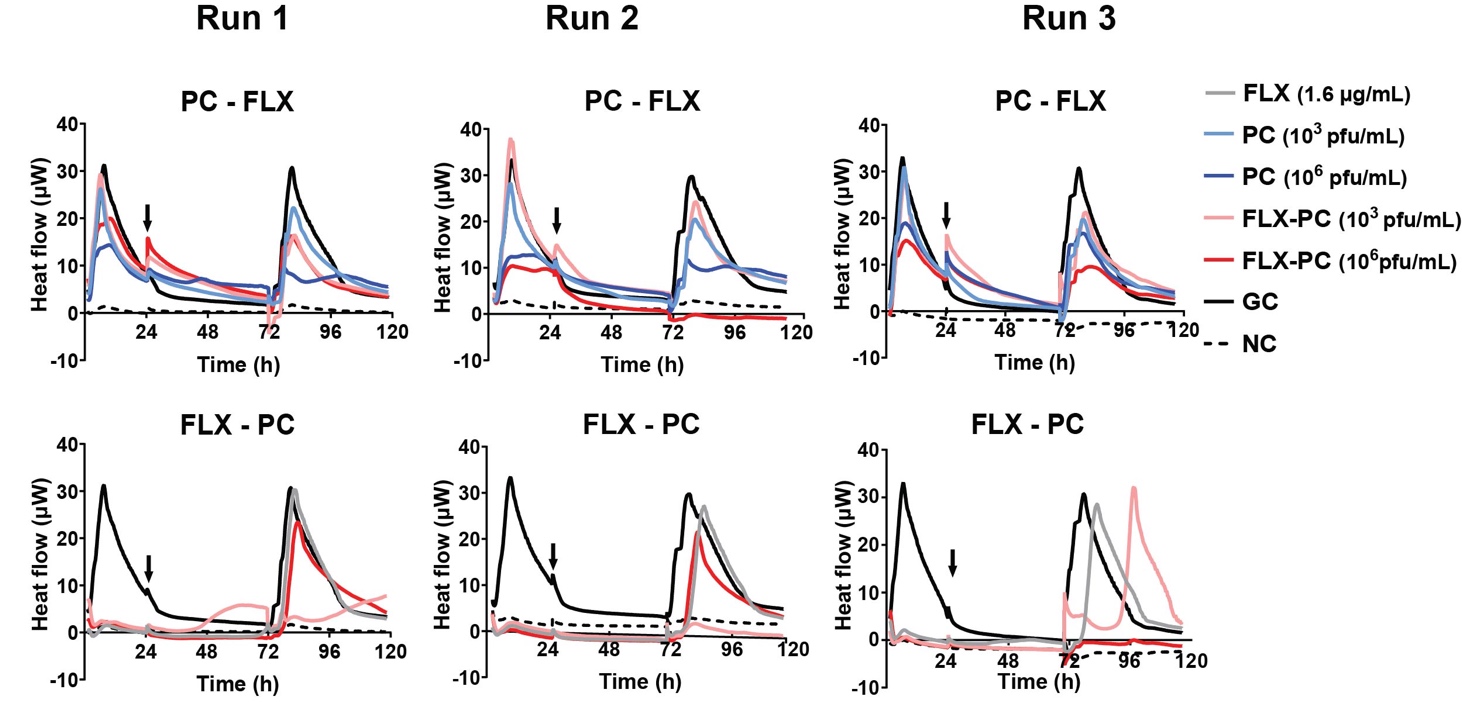


**Supplemental figure S4: Heat flow curves *S. aureus* biofilms after staggered treatment with the phage cocktail and flucloxacillin.** Flucloxacillin (FLX) at 1.6 µg/mL was added to the *S. aureus* biofilms twenty-four hours prior (FLX-PC) or after addition of the phage cocktail at 10^3^ or 10^6^ pfu/mL (PC-FLX). The addition of the second treatment is indicated with an arrow. Heat flow was measured in µW for 72 hours during treatment. The treatment was removed and regrowth was monitored for 24 hours. A medium only condition was included as negative control and an untreated condition as growth control. Three independent experiments are shown (Run 1-3).


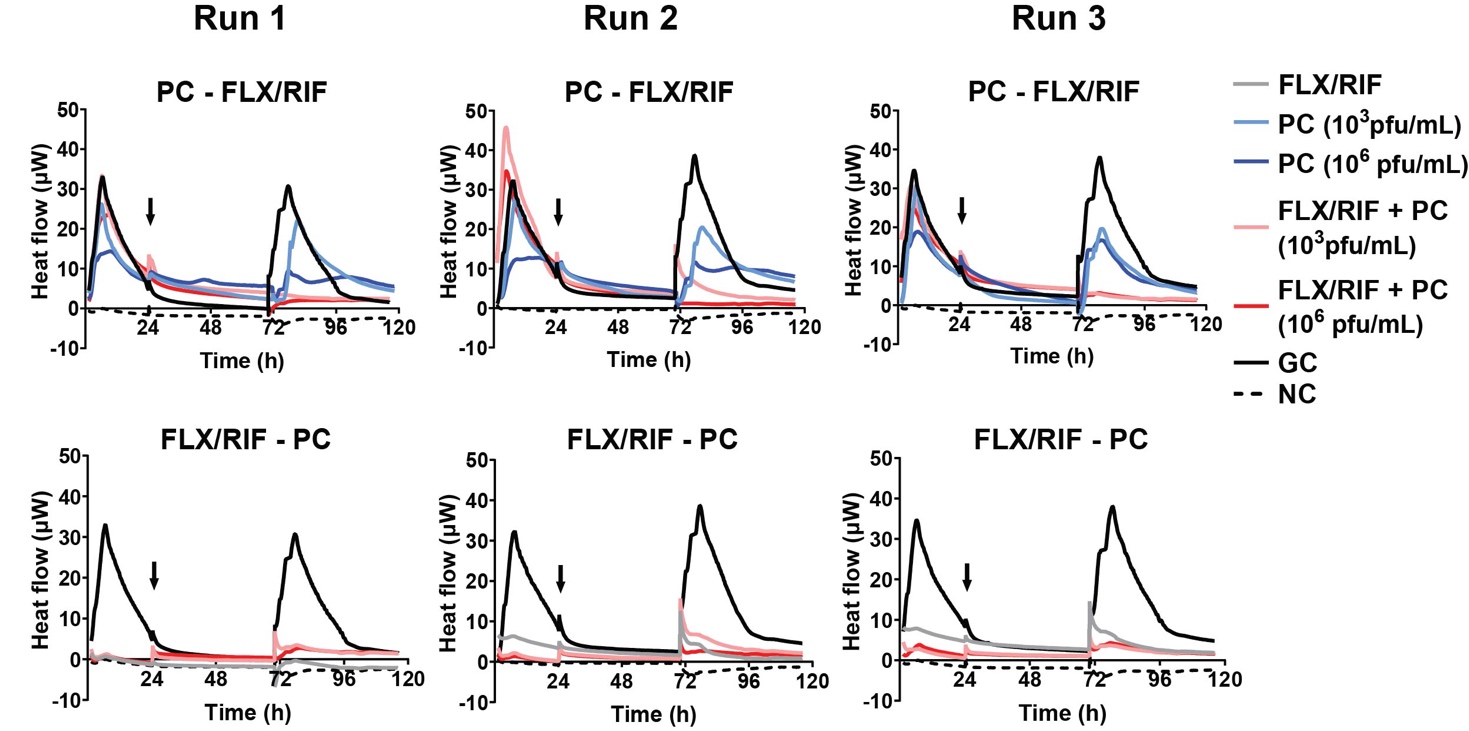


**Supplemental figure S5: Heat flow curves *S. aureus* biofilms after staggered treatment with the phage cocktail, flucloxacillin and rifampicin.** Flucloxacillin at 1.6 µg/mL was combined with 8 µg/mL rifampicin (FLX/RIF). These antibiotics were added to the *S. aureus* biofilms either twenty-four hours prior (FLX/RIF-PC) or after addition of the phage cocktail at 10^3^ or 10^6^ pfu/mL (PC-FLX/RIF). The addition of the second treatment is indicated with an arrow. Heat flow was measured in µW for 72 hours during treatment. The treatment was removed and regrowth was monitored for 24 hours. A medium only condition was included as negative control and an untreated condition as growth control. Three independent experiments are shown (Run 1-3).

**Supplemental Table S2: Coefficient of variance (CoV) was calculated over three replicates using**

$\boldsymbol{CoV=}\frac{\boldsymbol{standard. deviation}}{\boldsymbol{Mean of replicates}}$ **.** The CoV of both of the treatment of the SA-Ld biofilm with antibiotics and of regrowth phase is reported. Whenever negative or zero-values prevented CoV calculations, the standard deviation (S.DEV) was reported to represent variation between the three experiments.

| **Treatment** | **Concentration (ug/mL)** | **CoV**  **Treatment** | **S.DEV**  **Treatment** | **CoV**  **Regrowth** | **S.DEV**  **Regrowth** |
| --- | --- | --- | --- | --- | --- |
| **Cefuroxime** | 8 | 77.9 |  | 20.3 |  |
| **Cefotaxime** | 16 | 65.1 |  | 120.1 |  |
| **Gentamicin** | 16 | 138.3 |  | 37.8 |  |
| **Rifampicin** | 8 | 58.6 |  | 56.4 |  |
| **Clindamycin** | 8 | 38.7 |  | 59.1 |  |
| **Erythromycin** | 8 | 67.5 |  | 68.5 |  |
| **Linezolid** | 16 | 56.7 |  | 30.8 |  |
| **Fusidic acid** | 32 | 40.1 |  | 24.7 |  |
| **Co-trimoxazol** | 32 | 98.8 |  | 10.6 |  |
| **Doxycycline** | 4 | 6.8 |  | 24.5 |  |
| **Flucloxacillin** | 16 |  | 389.6 | 28.0 |  |
| **Flucloxacillin** | 1.6 | 73.0 |  | 11.5 |  |
| **Levofloxacin** | 8 | 101.8 |  |  | 27.9 |
| **Levofloxacin** | 0.8 | 105.9 |  | 84.9 |  |
| **Cefuroxime + Rifampicin** | 8 | 60.8 |  | 28.5 |  |
| **Cefotaxime + Rifampicin** | 16 | 39.2 |  | 36.8 |  |
| **Gentamicin + Rifampicin** | 16 | 16.7 |  | 34.0 |  |
| **Clindamycin + Rifampicin** | 8 | 116.3 |  | 19.9 |  |
| **Erythromycin + Rifampicin** | 8 | 82.3 |  | 47.3 |  |
| **Linezolid + Rifampicin** | 16 | 38.8 |  | 77.5 |  |
| **Fusidic acid + Rifampicin** | 32 | 78.6 |  | 30.4 |  |
| **Co-trimoxazol + Rifampicin** | 32 | 76.3 |  | 43.8 |  |
| **Doxycycline + Rifampicin** | 4 | 34.4 |  | 52.8 |  |
| **Flucloxacillin + Rifampicin** | 16 | 101.9 |  | 29.6 |  |
| **Flucloxacillin + Rifampicin** | 1.6 | 44.6 |  | 65.5 |  |
| **Levofloxacin + Rifampicin** | 8 | 99.8 |  | 156.1 |  |
| **Levofloxacin + Rifampicin** | 0.8 | 67.7 |  | 92.6 |  |
| **GC** | N/A | 81.4 |  | 14.8 |  |

**Supplemental Table S3: Coefficient of variance (CoV) was calculated over three replicates using**$\boldsymbol{CoV=}\frac{\boldsymbol{standard. deviation}}{\boldsymbol{Mean of replicates}}$**.** The CoV of both of the simultaneous and sequential treatment of the SA-Ld biofilm with flucloxacillin (FLX) combined with the phage cocktail (PC) and of regrowth phase. Whenever negative or zero-values prevented CoV calculations. the standard deviation (S.DEV) was reported to represent variation between the three experiments.

| **Simultaneous**  **Treatment** | **FLX (ug/mL)** | **PC (pfu/mL)** | **CoV**  **Treatment** | **S.DEV.**  **Treatment** | **CoV**  **Regrowth** | **S.DEV.**  **Regrowth** |
| --- | --- | --- | --- | --- | --- | --- |
| **Single treatments** | - | 10^3^ | 21.2 |  | 36.4 |  |
|  | - | 10^6^ | 19.4 |  | 12.6 |  |
|  | 0.25 | - | 18.4 |  | 16.6 |  |
|  | 1.6 | - | 79.9 |  | 86.7 |  |
| **FLX+PC** | 0.25 | 10^3^ | 6.4 |  | 12.6 |  |
|  | 0.25 | 10ˆ6 | 18.2 |  | 12.5 |  |
|  | 1.6 | 10^3^ |  | 3.0 | 5.7 |  |
|  | 1.6 | 10ˆ6 | 84.3 |  | 17.4 |  |
| **GC** | - | - | 5.6 |  | 5.4 |  |
| **Sequential**  **Treatment** | **FLX (ug/mL)** | **PC (pfu/mL)** | **CoV**  **Treatment** | **S.DEV.**  **Treatment** | **CoV**  **Regrowth** | **S.DEV.**  **Regrowth** |
| **Single treatments** | - | 10^3^ | 12.8 |  | 13.4 |  |
|  | - | 10^6^ | 4.1 |  | 12.0 |  |
|  | 1.6 | - | 45.6 |  | 8.5 |  |
| **PC - FLX** | 1.6 | 10^3^ | 36.0 |  | 25.0 |  |
|  | 1.6 | 10^6^ | 12.1 |  | 4.5 |  |
| **FLX - PC** | 1.6 | 10^3^ | 29.8 |  | 12.7 |  |
|  | 1.6 | 10^6^ | 4.0 |  | 7.7 |  |
| **GC** | - | - | 17.2 |  | 12.2 |  |

**Supplemental Table S4: Coefficient of variance (CoV) was calculated over three replicates using**$\boldsymbol{CoV=}\frac{\boldsymbol{standard. deviation}}{\boldsymbol{Mean of replicates}}$**.** The CoV of both of the simultaneous and sequential treatment of the SA-Ld biofilm with flucloxacillin (FLX) combined rifampicien (RIF) and the phage cocktail (PC) and of regrowth phase. Whenever negative or zero-values prevented CoV calculations. the standard deviation (S.DEV) was reported to represent variation between the three experiments.

| **Simultaneous**  **treatment** | **FLX**  **(ug/mL)** | **PC**  **(pfu/mL)** | **CoV**  **Treatment** | **S.DEV.**  **Treatment** | **CoV**  **Regrowth** | **S.DEV.**  **Regrowth** |
| --- | --- | --- | --- | --- | --- | --- |
| **Single Treatments** | - | 10^3^ | 20.4 |  | 16.8 |  |
|  | - | 10^6^ | 89.4 |  | 89.3 |  |
|  | 0.25 | - | 66.5 |  |  | 0.0 |
|  | 1.6 | - | 148.7 |  |  | 0.4 |
| **FLX-RIF+PC** | 0.25 | 10^3^ |  | 109.0 |  | 237.7 |
|  | 0.25 | 10^6^ |  | 78.5 |  | 0.6 |
|  | 1.6 | 10^3^ | 16.7 |  |  | 0.3 |
|  | 1.6 | 10^6^ | 162.6 |  |  | 1.6 |
| **GC** | - | - | 8.5 |  | 10.8 |  |
| **Sequential**  **treatment** | **FLX**  **(ug/mL)** | **PC (pfu/mL)** | **CoV**  **Treatment** | **S.DEV.**  **Treatment** | **CoV**  **Regrowth** | **S.DEV.**  **Regrowth** |
| **Single treatments** | - | 10^3^ | 0.0 |  | 0.0 |  |
|  | - | 10^6^ | 0.0 |  | 0.0 |  |
|  | 1.6 | - | 87.7 |  |  | 83.0 |
| **PC - FLX/RIF** | 1.6 | 10^3^ | 10.0 |  | 32.6 |  |
|  | 1.6 | 10^6^ | 10.9 |  | 28.7 |  |
| **FLX/RIF - PC** | 1.6 | 10^3^ | 67.9 |  | 36.1 |  |
|  | 1.6 | 10^6^ | 47.1 |  | 16.3 |  |
| **GC** | - | - | 15.5 |  | 19.6 |  |
